# Formyl peptide receptor 2 is an emerging modulator of inflammation in the liver

**DOI:** 10.1038/s12276-023-00941-1

**Published:** 2023-02-07

**Authors:** Chanbin Lee, Jinsol Han, Youngmi Jung

**Affiliations:** 1 Institute of Systems Biology, College of Natural Science, Pusan National University, Pusan, 46241 Republic of Korea; 2 Department of Integrated Biological Science, College of Natural Science, Pusan National University, Pusan, 46241 Republic of Korea; 3 Department of Biological Sciences, College of Natural Science, Pusan National University, Pusan, 46241 Republic of Korea

**Keywords:** Cell biology, Liver diseases

## Abstract

Formyl peptide receptors (FPRs), which are seven-membrane G-protein coupled receptors, recognize chemotactic signals to protect hosts from pathogenic infections and mediate inflammatory responses in the body. There are three isoforms of FPRs in humans—FPR1, FPR2, and FPR3—and they bind to N-formyl peptides, except FPR3, and to various endogenous agonists. Among FPR family members, FPR2 has a lower affinity for N-formyl peptides than FPR1 and binds with a wide range of endogenous or exogenous agonists. Thus, FPR2 is considered the most ambiguous member. Accumulating evidence has shown that FPR2 is involved in the host’s defense against bacterial infection and inflammation in liver diseases, such as nonalcoholic fatty liver disease, liver fibrosis, and liver cancer, suggesting the pathophysiological relevance of FPR2 to the liver. However, FPR2 has been shown to promote or suppress inflammation, depending on the type of FPR2-expressing cell and FPR2-bound ligands in the liver. Therefore, it is important to understand FPR2’s function per se and to elucidate the mechanism underlying immunomodulation initiated by ligand-activated FPR2 before suggesting FPR2 as a novel therapeutic agent for liver diseases. In this review, up-to-date knowledge of FPR2, with general information on the FPR family, is provided. We shed light on the dual action of FPR2 in the liver and discuss the hepatoprotective roles of FPR2 itself and FPR2 agonists in mediating anti-inflammatory responses.

## Liver disease: Inflammation-related receptor offers new drug target

Drugs directed at a receptor protein implicated in the body’s inflammatory responses could help protect the liver against fibrosis, cancer and other disorders. Youngmi Jung and Chanbin Lee from Pusan National University, South Korea, review how the formyl peptide receptor (FPR) family of proteins plays important roles in mediating immune responses to infection, injury and stress. One such family member, FPR2, is particularly active in certain liver diseases, including non-alcoholic fatty liver disease and hepatocellular carcinoma. However, there is laboratory evidence linking FPR2 expression both to the production and suppression of disease-provoking inflammatory molecules, depending on the type of cell and signaling molecules that interact with FPR2. A better understanding of this dual mechanism of action could help drug developers design FPR2-targeted therapies that protect against liver injury and fibrosis.

## Introduction

Inflammation is a host defense response to the loss of tissue homeostasis^1^. However, excessive inflammation is injurious to the body and can become a causative factor for a variety of diseases, significantly contributing to their pathophysiology^2^. The liver is continually exposed to pathogenic antigens from dietary and commensal metabolites delivered from the gastrointestinal tract through the blood^3^. In the liver, hepatic innate immune cells, such as Kupffer cells, dendritic cells, and natural killer cells, function as the first line of defense by clearing invading pathogens or toxins^4^. When the liver is exposed to chronic and/or severe damage, injured or dying hepatocytes release different signals to stimulate hepatic immune cells and to recruit neutrophils and monocyte-derived macrophages into the liver^5,6^. An inflammatory response initially promotes the wound-healing process to recover liver homeostasis, whereas unresolved persistent inflammation aggravates liver damage, leading to chronic liver disease^7^. This is why chronic inflammation is regarded as a common feature of liver diseases, such as nonalcoholic fatty liver disease (NAFLD), liver fibrosis, hepatitis C virus infection, and liver cancer^8^. Thus, modulating inflammation is important in preventing the progression of chronic liver disease.

When a host is attacked by invading pathogens or other factors, pattern recognition receptors (PRRs) play an important role in regulating the innate immune response as a nonspecific “sensor” for pathogen-associated molecular patterns or damage-related molecular patterns^9^. A member of the PRR class, formyl peptide receptors (FPRs), were first identified by Shiffmann et al. as receptors that recognize N-formyl peptides derived from degraded bacteria or damaged mitochondria^10^. FPRs belong to the seven transmembrane chemoattractant G-protein coupled receptors and consist of three members: FPR1, FPR2, and FPR3^11^. Among these, FPR2 is the most intriguing member because it creates a pro- or anti-inflammatory response according to its diverse ligands^12^. For instance, several polypeptides, such as serum amyloid A (SAA) and LL-37, elicit the proinflammatory action of Fpr2, while bioactive lipid molecules, including lipoxin A4 (LXA_4_) and resolvin D1 (RvD1), trigger an anti-inflammatory signaling cascade through FPR2^13^. A double-faceted action of FPR2 has also been shown regarding its impact on disease progression. FPR2 activation promotes the production of proinflammatory cytokines, which aggravate atherosclerosis and diabetic retinopathy^14,15^. In contrast, FPR2 is involved in suppressing disease progression in several organs/tissues, such as the liver, lung, brain, and kidney, by exerting anti-inflammatory effects. Oldekamp et al.^16^ reported that Fpr2 deficiency caused severe inflammation and higher mortality in mice with pneumococcal meningitis. LXA_4_-induced FPR2 activation decreased collagen accumulation and the expression of inflammatory cytokines by blocking TGF-β/Smad signaling in radiation-induced pulmonary fibrosis^17^. In the liver, FPR2 is related to disease progression. It was shown that FPR2 protected against bacterial hepatic infection by triggering neutrophil recruitment^18,19^. RvD1 treatment decreased hepatocyte death and mitochondrial dysfunction and increased the engulfment of dead and dying cells by macrophages, alleviating ischemia/reperfusion-induced liver injury^20,21^. In liver cancer, LXA_4_ enhanced cancer cell apoptosis and reduced HCC metastasis^22^. Lee et al.^23^ also demonstrated that FPR2 protected the liver from lipotoxicity and suppressed NAFLD progression. Contrary to these findings, however, there are also conflicting results. Chen et al.^24^ reported that Fpr2 deficiency attenuated high-fat diet (HFD)-induced obesity, insulin resistance, and macrophage infiltration into the liver. Therefore, it is necessary to focus on research on FPR2 and the development of therapeutics targeting FPR2. In this review, we summarize the general information on FPRs in human and experimental animal models. In addition, we focus on the pathophysiological role of FPR2 in inflammation-related liver disease and suggest its therapeutic potential.

## General information on the formyl peptide receptor family

FPR sequences are highly conserved in mammals, but their genetic characteristics, including their structure, gene number, and sequence, differ between humans and other species, such as rats, rabbits, guinea pigs, and mice^25,26^. Three FPRs have been identified in humans, namely, FPR1, FPR2 (referred to as formyl peptide receptor-like 1, FPRL1), and FPR3 (or FPRL2), and they are clustered on human chromosome 19q13.3^27^. They exhibit significant homology with each other in their amino acid sequences. FPR1 shares 69% identity with FPR2 and 56% homology with FPR3, while FPR2 and FPR3 share 83% identity in humans^13^. FPRs also share overlapping physiological roles, such as host defense against pathogens, regulation of inflammation, and clearance of damaged cells^28^. The murine Fpr family is most widely studied. Compared to human FPRs, the mouse Fpr gene family includes eight identified members: Fpr1, Fpr2 (Fpr-rs2), Fpr-rs1, Fpr-rs3, Fpr-rs4, Fpr-rs5, Fpr-rs6, and Fpr-rs7^29^. The entire mouse Fpr gene family is located within a cluster on mouse chromosome 17A3.2^29^. Among these mouse Fprs, the Fpr-rs5 gene is not encoded as a functional receptor despite the absence of typical pseudogene characteristics. The products of five other mouse Fpr genes, that is, Fpr-rs1, Fpr-rs3, Fpr-rs4, Fpr-rs6, and Fpr-rs7, are known chemosensory vomeronasal receptors in olfactory sensory neurons^30^. Mouse Fpr1 is considered a counterpart of human FPR1, and mouse Fpr2 functionally corresponds to human FPR2^31^. The mouse counterpart to human FPR3 has not been fully elucidated, but mouse Fpr-rs1 and Fpr2 partially replace the function of human FPR3 by interacting with F2L, a potent agonist of FPR3^32,33^. FPR1 and FPR2 are predominantly expressed in the membranes of myeloid cells, including leukocytes, monocytes, macrophages, natural killer cells, and dendritic cells^34,35^. They are also identified in nonhematopoietic cells. FPR1 has been detected in smooth muscle cells, lens epithelial cells, fibroblasts, astrocytes, and hepatocytes, and FPR2 has been observed in astrocytoma cells, epithelial cells, hepatocytes, microvascular endothelial cells, and neuroblastoma cells^28^. While FPR1 and FPR2 are found in diverse nonimmune cell types, FPR3 is more restricted to immune cells, such as monocyte-derived macrophages, mature dendritic cells, and tissue-specific macrophages in the colon, skin, and lung, but not in the liver^36^.

Although FPRs recognize N-formylate peptides, they have different binding affinities with these peptides according to their composition, origin, and length. *N*-Formylated peptides containing a methionine residue at the 5′ terminus have at least a 100-fold more potent affinity for FPR1 than identical nonformylated peptides^37^. Most gram-negative bacteria-derived formylated peptides interact more potently with FPR1 than FPR2^28^. For example, FPR1 binds to *Escherichia coli* (*E. coli*)-derived formyl-N-formyl-Met-Leu-Phe (fMLF) with an ~400-fold higher affinity than FPR2^38^. Formylated peptide-bound FPR1 provides a signal to recruit immune cells into the lesions of bacterial infection or injured tissue^39^. Although most formylated peptides bind to FPR1 with a higher binding affinity, some long formylated peptides, such as alpha-type phenol-soluble modulins (PSMα) and mitocryptide-2 (MCT-2), prefer to bind to FPR2 rather than to FPR1. PSMα produced by *Staphylococcus aureus* binds to FPR2 and stimulates neutrophil recruitment, the production of proinflammatory cytokines, and bacterial phagocytosis^40^. MCT-2, which originates from mitochondrial DNA-encoding cytochrome b, increases intracellular Ca^2+^ flux in neutrophils by interacting with FPR2^41^. In addition, FPR2 shows a more selective response to *N*-formylated peptides with a positive charge at the C-terminus, such as *N*-formyl-Met-Leu-Phe-Lys (fMLFK) and *N*-formyl-Met-Leu-Phe-Ile-Lys (fMLFIK), compared to *N*-formylated peptides with a negative charge on the C-terminus, such as *N*-formyl-Met-Leu-Phe-Glu (fMLFE) and fMLF^42^. FPR3 is nonresponsive to formylated peptides, despite a higher sequence identity between FPR1 and FPR3^33^ (Table 1).

**Table 1:** Characteristics of the FPR family.

	FPR1	FPR2	FPR3
Expressing cells			
Immune cells	Leukocytes, monocytes, macrophages, natural killer cells and dendritic cells		Monocyte-derived macrophages, mature dendritic cells and tissue-specific macrophages
Nonimmune cells	Smooth muscle cells, lens epithelial cells, fibroblasts, astrocytes and hepatocytes	Astrocytoma cells, epithelial cells, hepatocytes, microvascular endothelial cells, and neuroblastoma cells	–
Ligands			
N-formylated peptides	*N*-formylated peptides containing a methionine residue at the 5′ terminus including fMLF	PSMα, MCT-2 *N*-formylated peptides having a positive charge at the C-terminus, such as fMLFK and fMLFIK	–
Endogenous ligands	Cathepsin G	Amyloidogenic peptides, SAA, LXA_4_, RvD1, AnxA1	F2L

*fMLF* formyl-N-formyl-Met-Leu-Phe, *PSMα* alpha-type phenol-soluble modulin, *MCT-2* mitocryptide-2, *fMLFK* formyl-Met-Leu-Phe-Lys, *fMLFIK* formyl-Met-Leu-Phe-Ile-Lys, *SAA* serum amyloid A, *LXA*_*4*_ lipoxin A4, *RvD1* resolvin D1, *AnxA1* annexin A1.

## Double-edged action of FPR2 in the inflammatory response

Aside from formyl peptides, FPRs also recognize various types of endogenous lipids, nonformylated peptides, and proteins. Cathepsin G and F2L, which are endogenous ligands, are known to bind to FPR1 and FPR3, respectively, although few endogenous ligands for FPR1 and FPR3 have been identified. Cathepsin G, a neutrophil granule protein, has a weaker binding affinity to FPR1 than fMLF but enhances the chemotaxis of inflammatory cells by activating protein kinase Cζ, without inducing strong Ca influx in phagocytes^43^. FPR3, which exhibits a high affinity for endogenous F2L, is poorly understood^32^. Compared with FPR1 and FPR3, FPR2 has a broader range of ligands, including nonformylated peptides and proteins, lipid metabolites, and endogenous ligands^28^. Specifically, FPR2 could promote or inhibit the inflammatory response, depending on the type of ligand binding to it. For example, amyloidogenic peptide antimicrobial LL-37 stimulates a proinflammatory response^44,45^, and LXA_4_, RvD1, and annexin A1 (AnxA1) induce an anti-inflammatory response^46–48^. Herein, we have described the properties of FPR2 ligands and FPR2-mediated intracellular signaling involved in modulating the inflammatory response (Table 2).

**Table 2:** Summary of immunomodulating ligands of FPR2.

Ligand	Target cells/disease	Effects	Ref.
Proinflammatory ligands			
SAA	Phagocytes	Activated NF-κB signaling Increased expression of proinflammatory cytokines	^44^
	Neutrophils	Triggered ERK and AKT phosphorylation Suppressed apoptosis of neutrophils	^51^
	Monocytes	Facilitated recruitment of monocytes by stimulating NF-κB activation and AKT phosphorylation	^52^
Aβ_42_	Microglial cells	Phosphorylated ERK and increased production of proinflammatory cytokines	^55^
PrP_106-126_	Glial cells Monocytes	Elevated Ca2+ mobilization and increased production of proinflammatory cytokines	^57^
LL-37	Neutrophils/Eosinophils	Facilitated recruitment of immune cells and stimulated M1 polarization/Inhibited apoptosis of neutrophils	^45^^,^^58^
Anti-inflammatory ligands			
LXA_4_	Neutrophils	Inactivated NF-κB signaling and suppressed production of proinflammatory cytokines and proliferation	^63^^,^^64^
		Reduced p38 MAPK	^47^
	Macrophages	Enhanced phagocytosis of apoptotic cells	^62^
RvD1	Macrophages	Increased phagocytosis of apoptotic cells and recruitment of regulatory T cells	^46^^,^^66^
	Trophoblasts	Inactivated NF-κB signaling and suppressed production of proinflammatory cytokines	^67^
	Acute lung injury	Blocked IκBα degradation and NF-κB p65 nuclear translocation and production of proinflammatory cytokines	^68^
AnxA1	Monocytes	Increased anti-inflammatory cytokine IL-10 by stimulating p38 MAPK/MAPKAPK/Hsp27 signaling cascade	^48^
AnxA1/Ac2-26	Air-pouch model	Suppressed polymorphonuclear neutrophil recruitment and expression of MLP-1 α and prostaglandin E2	^69^
Ac2-26	Pneumococcal meningitis	Decreased granulocyte infiltration and proinflammatory responses	^70^

*SAA* serum amyloid A, *Aβ42* β-amyloid peptide 42, *PrP106-126* prion peptide fragment 106-126, *LXA4* lipoxin A4, *RvD1* resolvin D1, *AnxA1* annexin A1, *IκBα* nuclear factor of kappa light polypeptide gene enhancer in B cells inhibitor, alpha, *IL-10* interleukin-10, *MAPK* mitogen-activated protein kinase, *MLP-1α* macrophage inflammatory protein-1α.

### Fpr2 as a proinflammatory mediator

FPR2 recognizes amyloidogenic peptides, such as serum amyloid A (SAA), β-amyloid peptide 42 (Aβ_42_), and prion peptide fragment 106-126 (PrP_106-126_), which are associated with chronic inflammation. SAA is mainly produced by hepatocytes and macrophages and binds to extracellular loops I and II of FPR2^49,50^. SAA-stimulated FPR2 upregulates the amounts of proinflammatory cytokines and their receptors and promotes the survival of immune cells, enhancing the inflammatory response. He et al.^44^ reported that SAA bound to FPR2 activated nuclear factor-kappa B (NF-κB) signaling and increased the expression of proinflammatory IL-1, IL-6, IL-8, TNF-α and their receptors in myeloid-lineage phagocytes. It was shown that SAA interacting with FPR2 rapidly increased extracellular signal-regulated kinase (ERK) and AKT phosphorylation to induce myeloid cell leukemia-1 expression, a key regulator of neutrophil apoptosis, thus contributing to suppressed apoptosis in neutrophils^51^. SAA-activated FPR2 is also involved in facilitating the recruitment of monocytes by triggering ERK phosphorylation and NF-κB activation^52^. In addition, FPR2 senses neurotoxic Aβ_42_ and PrP_106-126_, which act as the main causative factors of Alzheimer’s disease by impacting the proinflammatory response. Aβ_42_ binds to extracellular loops and helices II, III, V, VI, and VII of FPR2 and is internalized in microglial cells^53,54^. In these cells, Aβ_42_ stimulates ERK phosphorylation and elevates the number of various cytokines and chemokines, including IL-1β, IL-6, interferon-γ, chemokine (C-C motif) ligand 2 (CCL2), chemokine (C-X-C motif) ligand (CXCL) 8, and CXCL10^55^. However, FPR2 also serves as a receptor for neuroprotective polypeptide humanin (HN), which acts as a competitive inhibitor of Aβ_42_ by occupying a region similar to the extracellular regions of FPR2 to which Aβ_42_ binds^54,56^. HN-bound FPR2 attenuates the fibrillary formation and cytotoxicity of Aβ_42_^56^. PrP_106-126_ interacting with FPR2 is internalized into glial cells, which are specialized macrophages in the central nervous system. Internalized PrP_106-126_ increases Ca^2+^ mobilization and the production of proinflammatory cytokines, such as IL-1β and IL-6, and induces the activation and migration of monocytic cells^57^. In addition, LL-37, which is an antimicrobial peptide derived from the breakdown of the neutrophil granule protein cathelicidin, is a proinflammatory ligand for FPR2. LL-37 bound with FPR2 induces the migration of neutrophils and eosinophils and stimulates differentiation of monocytes into the M1 phenotype of macrophages^58^. LL-37 also inhibits the apoptosis of neutrophils by blocking caspase-3 activity and upregulating anti-apoptotic Bcl-xL^45^.

### Fpr2 as an anti-inflammatory mediator

FPR2 is also capable of inhibiting inflammation by interacting with anti-inflammatory agonists, such as LXA_4_, RvD1 and AnxA1. LXA_4_ is a lipid metabolite derived from ω-6 arachidonic acid that retains anti-inflammatory properties^59^. LXA_4_ has a high binding affinity for FPR2 at extracellular loop III and the seventh transmembrane domain^49,60^. LXA_4_ binding to FPR2 leads to a conformational change in FPR2 to block the further binding of proinflammatory ligands such as Aβ_42_ and SAA^61^. The interaction between LXA_4_ and FPR2 activates various intracellular signaling pathways to relieve inflammation and results in a decrease in excessive neutrophil infiltration and the expression of proinflammatory cytokines and an increase in phagocytosis of apoptotic cells^62^. LXA_4_ binding to FPR2 inactivates NF-κB signaling in neutrophils and inhibits the production of proinflammatory cytokines and their proliferation^63,64^. LXA_4_ interacting with FPR2 also reduces the activity of p38 mitogen-activated protein kinase (MAPK) and elevates the levels of nuclear factor erythroid 2-related factor 2 and peroxisome proliferator-activated receptor gamma (PPARγ) which are the factors alleviating the expression of proinflammatory genes^47^. Maderna et al.^62^ reported that LXA_4_ stimulated macrophages through FPR2 and increased phagocytosis of apoptotic cells, attenuating the inflammatory response. FPR2-expressing macrophages significantly eliminated apoptotic cells under the condition of LXA_4_ stimulation, whereas Fpr2-deficient macrophages rarely did, even though LXA_4_ treatment was given^62^.

RvD1, a derivative of docosahexaenoic acid, also plays an important role in ameliorating inflammation by interacting with FPR2, although the binding region of RvD1 on FPR2 remains unclear^65^. RvD1 increases apoptotic cell clearance by macrophages and decreases the migration of inflammatory cells through FPR2^46,66^. Luo et al.^46^ showed that RvD1 treatment enhanced the phagocytosis of dying cells to stimulate TGF-β expression in macrophages and induced the recruitment of regulatory T cells to promote inflammation resolution in autoimmune neuritis. RvD1 binding to FPR2 inactivates the NF-κB pathway in a PPARγ-dependent manner and reduces the secretion of proinflammatory cytokines, such as IL-1β, IL-6, and TNF-α, in chorioamnionitis^67^. In an experimental animal model of acute lung injury, FPR2 activated by RvD1 suppressed IκBα degradation and NF-κB p65 nuclear translocation and downregulated the proinflammatory cytokines IL-1β, IL-6, and TNF-α^68^.

In addition to bioactive lipid mediators, AnxA1 and its derived peptide, Ac2-26, bind to FPR2 and are involved in the anti-inflammatory response. Although a high concentration of AnxA1 (100–200 μM) activates FPR1 and has a proinflammatory effect, a low concentration of AnxA1 (10–20 μM) binds to FPR2 and leads to an anti-inflammatory response^69^. In monocytes, AnxA1 binding to FPR2 stimulates the p38 MAPK/MAPKAPK/HSP27 signaling cascade and increases the production of the anti-inflammatory cytokine IL-10^48^. Pettetti et al.^69^ found that both Ac2-26 and AnxA1 interacting directly with FPR2 limited polymorphonuclear neutrophil recruitment to inflammatory loci and suppressed the expression of macrophage inflammatory protein-1α and prostaglandin E2, leading to a decrease in dermal inflammation in an air-pouch model. Ac2-26 was also shown to alleviate granulocyte infiltration and proinflammatory responses during pneumococcal meningitis by interacting with FPR2^70^. Therefore, these ligands bound to FPR2 with anti-inflammatory action have been targeted to develop treatments for diseases with severe inflammation.

## Focusing on FPR2 in the liver

Because chronic inflammation is considered a common feature of liver disease, the regulation of inflammation is an important therapeutic strategy for preventing disease progression^8^. According to the evidence obtained thus far, FPR2 induces opposing inflammatory responses, promoting or inhibiting inflammation, but it is clear that FPR2 is involved in the inflammatory response in the liver. Given the limited information on FPR2 in the liver and the fact that FPR2 ligands causing anti-inflammatory action have therapeutic potential for chronic liver disease, it is necessary to determine and understand the pathogenetic action of FPR2 in liver disease to determine its effective clinical approaches. Herein, we summarized and discussed the accumulated evidence for FPR2’s function in the liver to provide basic knowledge and suggest the research needed on FPR2 in the liver.

### FPR2 influences neutrophil recruitment in hepatic infection by bacteria

As one of the FPR members, FPR2 also participates in the host’s immune response against bacterial infection by recognizing pathogen-derived danger signals or antibacterial host responses. Several papers have reported the essential role of FPR2 in host defense in cases of infected liver. When mice were infected with bacteria, *Fpr2*-deficient mice had a distinctly higher mortality rate than WT mice^16,19,71^. Liu et al.^19^ demonstrated that FPR2 activation facilitated H_2_O_2_ production in neutrophils to remove bacteria by increasing ERK1/2 phosphorylation. In addition, neutrophils expressing *Fpr2* rapidly migrated into the infected liver by reacting with *Listeria monocytogenes*-released chemotactic signals, whereas neutrophils lacking *Fpr2* failed to do so^19^. FPR2 also senses the *E. coli*-derived chemotactic peptide fMLF in murine neutrophils^71^. In addition, when neutrophils were treated with WRW4, an FPR2 antagonist, these cells lost their chemotactic ability for fMLF^71^. Sun et al.^18^ have reported that *Fpr2*-deficient mice had impaired infiltration to the infected site and were more susceptible to *Streptococcus agalactiae* (also known as group B streptococcus; GBS) infection than WT mice. In their research, FPR2 upregulated CXCL1 and CXCL2 during GBS and indirectly promoted neutrophil recruitment to the liver, rather than directly stimulating the migration of these cells into the liver. These findings clearly present a crucial role for FPR2 in neutrophil recruitment in response to bacterial infection.

### FPR2 is associated with the inflammatory response in liver disease

FPR2 has anti-inflammatory properties and attenuates the progression of liver disease, as supported by the results obtained from experiments with *Fpr2*-depleted mice. Giebeler et al.^72^ reported that a ubiquitous deficiency of *Fpr2* enhanced the infiltration of immune cells with increased levels of proinflammatory genes, such as IL-6, TNF-α, CXCL1, TLR2, and TLR4, in the livers of liposaccharide-injured mice. A higher number of TUNEL-positive apoptotic cells and a lower number of Ki-67-positive proliferating cells were also observed in mice with systemic *Fpr2* deletion than in WT mice. Recently, Lee et al.^23^ demonstrated that FPR2 is involved in preventing the development and/or progression of NAFLD. NAFLD shows a different prevalence based on sex in that men have a higher prevalence than women before age 50^73^. However, its prevalence increases and becomes higher in women than in men after age 50^74^. The authors showed significantly higher expression of FPR2 in the hepatocytes and livers of WT female mice than in WT male mice and that this female-specific FPR2 in hepatocytes protected these cells from lipotoxicity, contributing to the resistance to NAFLD development and severity in female mice. These findings suggest that FPR2 is involved in regulating sex-specific responses to nonalcoholic injuries. Contrary to the results of this study, Chen et al.^24^ reported that ubiquitous *Fpr2* deletion attenuated insulin resistance and hepatic steatosis in HFD-fed mice. It was also shown that *Fpr2* deficiency reduced macrophage recruitment and the production of serum IL-6, TNF-α, and CCL2 by inhibiting M1 polarization of macrophages. The hepatoprotective effect caused by a lack of *Fpr2* disappeared when FPR2 expression was induced in immune cells. In addition, myeloid-specific deletion of *Fpr2* alleviated diet-induced liver damage, insulin resistance, and macrophage infiltration. These two papers suggest the contrasting effects of FPR2 in NAFLD and focus on different types of liver cells expressing FPR2: hepatocytes and immune cells. The NAFLD animal models employed in the two groups were also different. Compared to a HFD, a choline-deficient, L-amino acid-defined high-fat diet (CDAHFD) induces more excessive lipid accumulation in the liver by restricting lipid secretion due to choline deficiency, resulting in more severe liver damage, such as inflammation and fibrosis, during the same period of treatment as the HFD^75^. However, the CDAHFD rarely induces adiposity, body weight gain, or peripheral insulin sensitivity because the diet contains minimal methionine to maintain visceral fat mass, whereas a HFD leads to an increase in body weight and insulin resistance^76,77^. Although they used different animal models of NAFLD, Lee et al. investigated FPR2 action in a nonalcoholic steatohepatitis (NASH)-like model induced by CDAHFD feeding for a longer time compared to the HFD treatment period, and Chen’s group examined FPR2’s role in NAFL, a milder disease than NASH. Namely, the action of FPR2 seems to have been analyzed at different stages of disease progression. In addition, the expression of hepatic FPR2 hardly changed during either HFD or CDAHFD feeding. According to Lee’s group, hepatic FPR2 expression was significantly lower in males than in females, and FPR2 in hepatocytes was even absent in healthy male mice, whereas it was distinctly present in hepatocytes from healthy female mice. The hepatoprotective effect of FPR2 observed in female mice could occur in male mice by artificially inducing FPR2. These findings indicate that the hepatic function of FPR2 is more influential in female mice than in male mice and suggest the need to assess the presence or absence of *Fpr2* expression in the liver before examining its role in the liver. Although Chen’s group found that ubiquitous deletion of *Fpr2* reduced the amounts of inflammatory cytokines in serum, the levels of inflammatory markers in the liver itself did not differ significantly between WT and *Fpr2*-deficient male mice during HFD feeding. Rather, *Fpr2* deficiency significantly downregulated inflammatory markers in WAT and muscle, but not liver. Given that crosstalk among liver, fat, and muscle plays an important role in the pathogenesis of NAFLD^78^, the mitigated inflammation in WAT and muscle caused by *Fpr2* removal seems to lead to decreased inflammation in the livers of *Fpr2*-deficient male mice. However, the functions of FPR2 in hepatocytes and immune cells in the liver remain to be elucidated. Therefore, additional studies are needed to determine the cell-specific function, transcriptional regulation, and intracellular signal cascades impacted by FPR2 in hepatocytes and inflammatory cells.

### Ligands binding to FPR2 exert hepatoprotective action

**Fig. 1:**
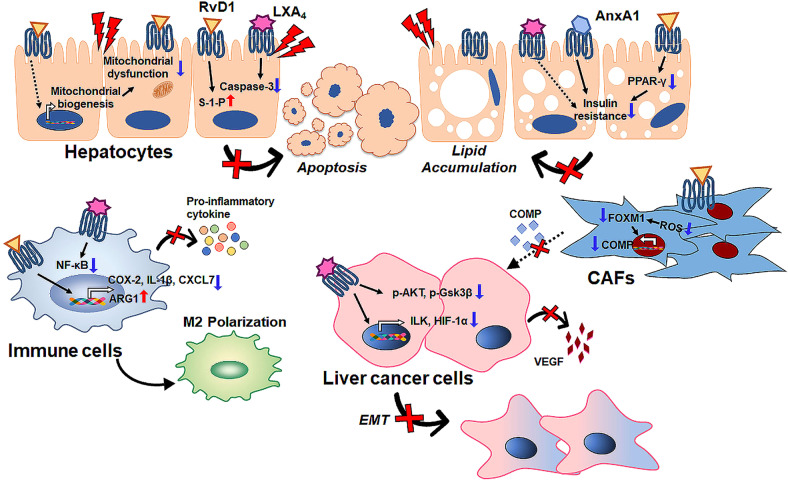
A schematic depicting the hepatoprotective effects of ligand-mediated FPR2 activation. Anti-inflammatory agonists have therapeutic potential for chronic liver disease by activating FPR2. In ischemia/reperfusion (IR)-induced liver, resolvin D1 (RvD1) treatment improves sphingosine-1-phosphate (S-1-P) activity and mitochondrial dysfunction and reduces hepatocyte apoptosis. Lipoxin A4 (LXA4) also inhibits hepatocyte apoptosis by suppressing caspase-3 action in the damaged liver. FPR2 bound with these anti-inflammatory ligands alleviates lipotoxic stress in hepatocytes. Annexin A1 increases insulin receptor substrate 1 signaling and decreases hepatic lipid contents. RvD1 binding to FPR2 lowers hepatic lipid accumulation by downregulating peroxisome proliferator-activated receptor gamma (PPARγ). In immune cells, both LXA4 and RvD1 reduce inflammation by interacting with FPR2. RvD1 treatment decreases the levels of M1 polarization-related genes such as cyclooxygenase-2 (COX-2), interleukin-1 beta (IL-1β), and C-C chemokine receptor type 7 (CXCL7) and increases the expression of the M2 polarization-related gene arginase 1 (ARG1). In liver cancer, RvD1 triggers the FPR2/ROS/FOXM1 signaling pathway and blocks the secretion of cartilage oligomeric matrix protein (COMP) in cancer-associated fibroblasts (CAFs), suppressing epithelial-mesenchymal transition (EMT) and the gain of stemness features in HCC cells. In addition, LXA4 alleviates the levels of integrin-linked kinase (ILK), hypoxia-inducible factor-1α (HIF-1α), and vascular endothelial growth factor (VEGF) and the phosphorylation of Akt and GSK3β and suppresses cancer cell proliferation, EMT and angiogenesis.

Although FPR2 is known to regulate disease progression as an immune modulator, based on data obtained from *Fpr2* knockout animals, its function also depends on which ligands are bound and which cellular signaling systems are activated. Several studies have reported that the anti-inflammatory ligands of FPR2 alleviate liver disease (Fig. 1). Proresolving agonists increased hepatocyte survival in the damaged liver. RvD1 treatment reduced hepatocyte apoptosis and serum levels of inflammatory cytokines, such as TNF-α, IL-6, IL-10, and monocyte chemoattractant protein-1, in a D-galactosamine (D-GalN)-sensitized mouse endotoxin shock model^79^. Several papers have reported that RvD1 protects the liver against ischemia/reperfusion (IR)-induced injury. Supplementation with RvD1 reduced hepatocyte necrosis in IR-injured livers by activating sphingosine-1-phosphate, which promotes cell growth and survival and inhibits apoptosis^20^. RvD1 also improved mitochondrial dysfunction by increasing the protein expression associated with mitochondrial biogenesis in IR-damaged liver^21^. Kang et al.^80^ revealed that RvD1-activated FPR2 triggered the M2 polarization of macrophages and enhanced efferocytosis, ameliorating IR-induced liver damage. In addition, LXA_4_ treatment was shown to suppress the activities of caspase-3 and nuclear NF-κB in both hepatocytes and Kupffer cells, leading to a reduction in hepatocyte death in mice with acute liver failure caused by D-GalN/LPS^81^.

Recently, the action of these ligands binding to FPR2 has been proven in NAFLD. Börgeson et al.^82^ demonstrated that LXA_4_ attenuated HFD-induced liver injury by reducing hepatic triglyceride accumulation in mice. In obese mice with steatohepatitis, RvD1 treatment decreased the intrahepatic lipid content and insulin resistance by decreasing the expression of PPARγ and increasing the level of circulating adiponectin^83^. RvD1 also mitigated hepatic inflammation by downregulating M1 polarization markers, such as cyclooxygenase-2, IL-1β, and C-C chemokine receptor type 7, and upregulating the M2 marker arginase 1, thereby reinforcing the effect of calorie restriction^83^. Li et al.^84^ demonstrated that RvD1 alleviated NASH progression by decreasing the TLR4-MyD88‐mediated NF‐κB and MAPK signaling pathways and increasing the expression of nuclear factor erythroid-2-related factor 2, a transcription factor for the antioxidant genes heme oxygenase-1, nicotinamide-adenine dinucleotide phosphate, and quinone oxidoreductase-1. AnxA1 treatment attenuated the development of obesity and insulin resistance by improving insulin receptor substrate 1 signaling in HFD-fed mice^85^. It was also reported that a deficiency of endogenous AnxA1 enhanced the activation of liver macrophages and fibrosis in mice with NASH^86^. However, because a high concentration of AnxA1 could induce a proinflammatory response via FPR1 rather than FPR2, further study of the AnxA1-stimulated receptor type subsequent to the activated signaling pathway is necessary to determine the action of AnxA1 in NAFLD.

The antitumor action of FPR2 has been reported by a few papers that show that ligand-activated FPR2 inhibits both the proliferation of HCC cells and their acquisition of stemness features. In the tumor microenvironment, hepatic stellate cells (HSCs) serve as cancer-associated fibroblasts and release cartilage oligomeric matrix protein, enhancing the invasion and metastasis of HCC^87^. Sun et al.^87^ reported that RvD1 reduced HSC-derived COMP paracrine signaling through the FPR2/ROS/Forkhead Box M1 signaling pathway and inhibited the epithelial-mesenchymal transition (EMT) and stemness of HCC cells. LXA_4_ treatment downregulated both the expression of integrin-linked kinase expression and the phosphorylation of AKT and GSK3β and interrupted EMT, migration and metastasis of HCC in an in vitro model^22^. LXA_4_ treatment also increased the apoptosis of liver cancer cells and blocked their proliferation by effectively blocking proliferation signals from macrophages^88^. In H22 cells, a mouse hepatocellular carcinoma cell line, exogenous LXA_4_ exerted an antiangiogenic effect by suppressing the production of vascular endothelial growth factor and reducing hypoxia-inducible factor-1α levels^89^. FPR2 agonists have consistently been shown to suppress liver cancer progression, suggesting that FPR2 agonists may represent potential candidates for the treatment of liver cancer. However, data demonstrating the action of FPR2 agonists in preventing HCC progression are limited, and further study is required to support their role in HCC before targeting them as therapeutics for liver cancer.

## Future perspectives and conclusion

FPR2 modulates inflammation, hepatocellular death, and lipid accumulation and inhibits the invasion and metastasis of liver cancer cells, contributing to controlling the progression of chronic liver disease. In the damaged liver, FPR2 is involved in the inflammatory response and promotes or suppresses disease progression. However, growing evidence indicates that FPR2 functions as an anti-inflammatory modulator to attenuate the progression of liver disease. Hence, effective synthetic agonists and analogs of proresolving FPR2 ligands, such as Trp-Lys-Tyr-Met-Val-D-Met (WKYMVm) and BML-111, have been developed that focus on the anti-inflammatory effect of FPR2. The synthetic peptide WKYMVm is a strong agonist for FPR2, with higher affinity than other FPRs, and has anti-fibrotic and regenerative effects in fibrotic liver^90^. Jun et al.^90^ found that WKYMVm treatment decreased the expression of fibrotic markers, α-smooth muscle actin, and type I collagen and increased the levels of angiogenetic factors, vascular endothelial growth factor (VEGF), and VEGF receptors in a bile duct ligation model. WKYMVm also improved hepatocyte proliferation through IL-6/GP130/STAT3 signaling. Recently, the same group reported that WKYMVm improves the proangiogenic, regenerative, and anti-fibrotic abilities of placenta-derived mesenchymal stem cells (PD-MSCs) via FPR2, and combined treatment of WKYMVm with PD-MSCs significantly reduces hepatic damage and improves hepatocyte proliferation in rats with cholestatic liver compared to PD-MSCs alone^91^. However, WKYMVm also binds to a receptor, namely, the hepatocyte growth factor receptor (HGFR), other than FPR2. Cattaneo et al.^92^ demonstrated that WKYMVm induced activation of HGFR and that activated HGFR phosphorylated the Y705 and S727 residues of STAT3 to facilitate nuclear translocation of phosphorylated STAT3, which acted as a transcription factor in human prostate epithelial cells. Jun et al. have shown that WKYMVm suppresses HSC activation and liver fibrosis but did not show whether HSCs express FPR2 in response to WKYMVm; this is still unclear. A few reports have shown that FPR2 is rarely expressed by HSCs^23,93^. It is possible that WKYMVm interacting with other receptors, such as HGFR expressed by HSCs, directly regulates HSC activation or binds to FPR2 expressed by other types of cells, which indirectly impacts HSC activation. Thus, it is necessary to first confirm the expression of FPR2 in HSCs, and further studies are needed to determine how WKYMVm influences HSC activation.

BML-111, an analog of LXA_4,_ has been shown to reduce articular neutrophil accumulation through FPR2. El-Agamy et al.^94^ revealed that BML-111 treatment lowered the degree of hepatocellular necrosis, oxidative stress, and inflammation in acetaminophen-induced acute liver injury. BML-111 also suppressed HCC progression by inhibiting proliferation, migration, EMT, and metastasis^22^.

Growing evidence indicates that FPR2 exerts anti-inflammatory action and attenuates liver injury. However, a few studies have demonstrated the pathogenic role of FPR2 in accelerating the disease state. FPR2 was even shown to promote or inhibit NAFLD, depending on the types of cells expressing it. Because FPR2 has shown opposing effects on hepatocytes and inflammatory cells under lipotoxic conditions, it is important to study the cell type-specific role of FPR2 in the liver. In addition, it is first necessary to clarify FPR2 expression before defining its function in the liver. Therefore, an understanding of the sophisticated mechanism of FPR2 activation may help to develop treatments that selectively promote the hepatoprotective effect of FPR2, which may be a promising strategy against liver disease.
